# TIP30 regulates lipid metabolism in hepatocellular carcinoma by regulating SREBP1 through the Akt/mTOR signaling pathway

**DOI:** 10.1038/oncsis.2017.49

**Published:** 2017-06-12

**Authors:** F Yin, G Sharen, F Yuan, Y Peng, R Chen, X Zhou, H Wei, B Li, W Jing, J Zhao

**Affiliations:** 1 PLA General Hospital Cancer Center Key Lab, PLA Postgraduate School of Medicine, Beijing, China; 2 Molecular Pathology Laboratory, College of Basic Medicine, Inner Mongolia Medical University, Hohhot, China; 3 Department of Orthopedics, PLA General Hospital, Beijing, China; 4 International Joint Cancer Institute, The Second Military Medical University, Shanghai, China; 5 Changhai Hospital, The Second Military Medical University, Shanghai, China; 6 Shanghai University Of Medicine & Health Sciences, Shanghai, China

## Abstract

Lipid reprogramming has been considered as a crucial characteristic in hepatocellular carcinoma (HCC) initiation and progression. However, detailed molecular mechanisms have yet to be clearly defined. Here, we examined the effects of tumor suppressor TIP30 on the regulation of HCC lipid metabolism. We found that decreased TIP30 expression leads to elevated fatty acid synthesis and enhanced levels of lipogenic enzymes SCD and FASN in HCC cells. Moreover, SREBP1 is one of the key transcription factors regulating liver lipid metabolism, and TIP30 deficiency significantly increased SREBP1 expression and nuclear accumulation. Small interfering RNAs targeting SREBP1 could reverse fatty acid synthesis induced by TIP30 deficiency. Furthermore, downregulating TIP30 activated the Akt/mTOR signaling pathway to upregulate SREBP1 expression, which promoted lipid metabolism by activating gene transcription of lipogenesis, including *fasn* and *scd.* We also showed that TIP30 deficiency-regulated lipid metabolism promoted proliferation of HCC cells. Clinically, our data revealed that TIP30 expression significantly correlated with SREBP1 in patients with HCC and that a combination of TIP30 and SREBP1 is a powerful predictor of HCC prognosis. Together, our data suggested a novel function of TIP30 in HCC progression and indicate that TIP30 regulation of SREBP1 may represent a novel target for HCC treatment.

## Introduction

The morbidity and mortality of hepatocellular carcinoma (HCC) ranks top5 and top3 respectively among common malignant tumors worldwide. The poor outcomes of HCC patients are mainly due to high recurrence of HCC after surgery and resistance to chemotherapy.^1^ Meanwhile, sorafenib was used as first line targeted drugs in treating advanced HCC, which can only prolongs the survival period of less than 3 months.^2^ Consequently, to elaborate the pathogenesis and progression of HCC and to develop new therapeutic strategies seem extremely crucial.

Major risk factors of HCC are viral hepatitis, exposure to hepatotoxins and alcohol abuse, whereas recent clinic and epidemiology researches indicate nonalcoholic fatty liver disease (NAFLD) increases HCC incidence.^3^ Recently, metabolic reprogramming, especially lipid metabolism alteration, is considered to be the initiating factor of tumor occurrence and progression. Continuous *de novo* cholesterogenesis and lipogenesis are frequently activated in tumors for providing extra lipids and lipid precursors during rapid cell proliferation.^4^ Several key enzymes have been identified to promote *de novo* lipid synthesis, including fatty acid synthase (FASN), stearoyl-CoA desaturase (SCD) and acetyl-CoA carboxylase (ACC).^5^ However, detailed mechanisms of abnormal lipid metabolism have not yet been comprehensively identified during HCC progression.

TIP30, namely HTATIP2 or CC3, was firstly discovered in small cell lung carcinoma, using differential analyses between highly metastatic human variant cells and less metastatic classic cells.^6^ Subsequently, TIP30 was found to be downregulated in various tumors and is considered to be a tumor suppressor due to its pro-apoptotic activity and its anti-metastatic and anti-angiogenic capacities.^7, 8, 9, 10, 11^ Our previous research reported that TIP30-regulated tumor metastasis and chemoresistance in various cancers.^12, 13, 14, 15, 16, 17, 18^ Moreover, we also found downregulated TIP30 induces epithelial–mesenchymal transition in HCC and pancreatic cancer.^19, 20^

Considering the crucial role of lipid metabolic reprogramming in cancer development, identifying new molecules and pathways that are involved in this process is vital. Recently, TIP30 has been preliminarily revealed to affect fatty acid storage and oxidation in hepatocytes^21^ and we will extensively investigate the role of TIP30 in lipid metabolism deregulation of HCC. We indicate decreased TIP30 promotes lipid metabolism via Akt/mTOR/SREBP1 signaling and that the combination of TIP30 and SREBP1 is an effective predictor for HCC prognosis.

## Results

### TIP30 is a negative regulator of lipid metabolism in HCC cells

**Figure 1:**
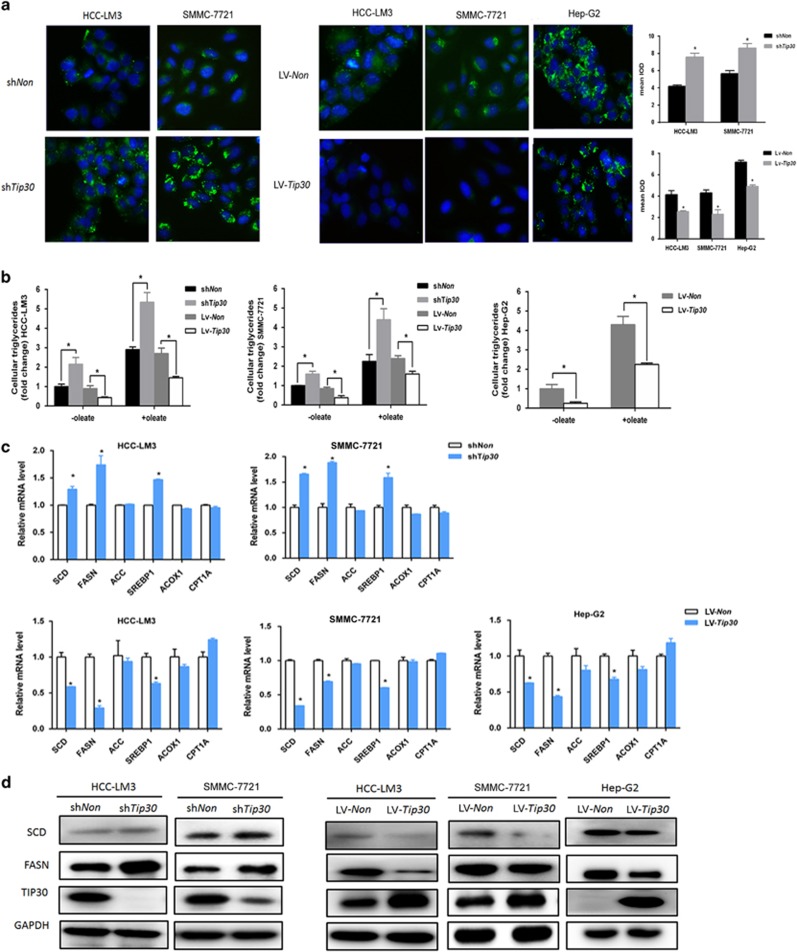
TIP30 negatively regulates lipid metabolism of hepatocellular cancer cell lines. (**a**) HCC cells (HCC-LM3, SMMC-7721 and HepG2) were infected with lentivirus to knockdown or overexpress TIP30. BODIPY 493/503 (green staining) was used to stain neutral lipids of each cell. DAPI (blue staining) was used to stain nuclear. (magnification, × 200). For quantification of the mean integrated optical density (IOD) of BODIPY staining, Image J software was applied and values were analyzed with unpaired *t*-test. (**b**) Intracellular triglyceride levels were detected in each cell, with or without lipid accumulation inducer, oleate (0.05 mm), added in the culture medium. (**c**) qRT–PCR methods were applied to test mRNA levels of lipogenic enzymes (ACC, FASN, SCD and SREBP1) and fatty acid oxidation enzymes (ACOX1 and CPT1A) in the indicated cells. (**d**) Western blot was applied to detect the SCD and FASN expression in each cell. Protein levels of TIP30 were also detected to determine the efficiency of lentivirus infection. *P<0.05.

To determine whether TIP30 regulates lipid metabolism of HCC cells, microarray analysis was firstly applied for comparing gene expression profiles of HCC-LM3 infected with sh*Non* or sh*Tip30*. 837 genes were differentially expressed upon TIP30 knockdown (fold change ⩾2, *P*<0.05). Through GO and KEGG enrichment analysis, these genes were enriched in several biological pathways. The results indicated that genes that significantly correlated to TIP30 expression were involved in fatty acid metabolism (Supplementary Figure 1A), which suggested the important role of TIP30 involved in lipid metabolism regulation. Furthermore, effects of TIP30 on cellular lipid levels were explored in HCC-LM3, SMMC-7721 and HepG2 cell lines using the lipophilic dye BODIPY 493/503. We employed lentiviruses to knockdown or overexpress TIP30 expression in HCC cell lines, and the lentiviral infection efficiency is showed in Figure 1d. We observed that silencing TIP30 led to increased levels of neutral lipid staining in HCC-LM3 and SMMC-7721 cells, whereas the staining was significantly decreased in TIP30-overexpressed cells (Figure 1a). Similar results were obtained when the lipid accumulation inducer oleate was added to the culture medium (Supplementary Figure 1B). Consistently, intracellular triglyceride levels were further determined to support the negative effects of TIP30 on lipid metabolism regulation (Figure 1b).

To well explain the mechanisms of TIP30 regulating *de novo* lipid synthesis, we analyzed lipogenesis-related enzymes (FASN, SCD, and ACC) levels of HCC cells with different TIP30 expressions. Results demonstrated both the mRNA and protein levels of SCD and FASN notablely elevated in TIP30-depleted cells, whereas overexpression of TIP30 reduced SCD and FASN levels comparing to control (Figures 1c and d). However, ACC expression remained unchanged with TIP30 knockdown or overexpression (Figure 1c). As fatty acid oxidation (FAO) played an important role in lipid metabolism reprogramming in several types of cancer, we also analyzed the levels of critical FAO-related factors, including CPT1A and ACOX1. However, TIP30 exerted no effects on these two oxidative enzymes in HCC cells (Figure 1c). Taken together, our research indicates TIP30 can regulate *de novo* fatty acid synthesis of HCC cells.

### SREBP1 is essential for TIP30 deficiency-mediated lipogenesis-promoting effects

**Figure 2:**
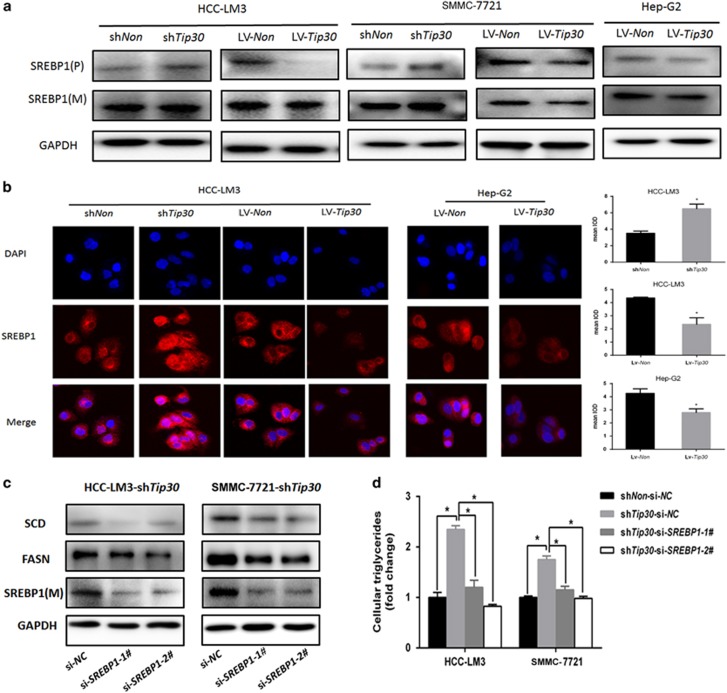
SREBP1 is essential for TIP30 deficiency-mediated lipogenesis-promoting effects. (**a**) Western blot was performed for detecting cytosolic precursor (P) and nucleic mature (M) forms of SREBP1 in indicated HCC cells lysates. (**b**) Results of immunofluorescence analysis were showed in indicated HCC cells. Red staining represented SREBP1 protein. DAPI (blue staining) was used to stain nuclear. (magnification, × 200) For quantification of the mean integrated optical density (IOD) of BODIPY staining, Image J software was applied and values were analyzed with unpaired *t-*test. (**c**) Indicated protein levels were detected in HCC-LM3-*shTip30* and SMMC-7721-*shTip30* cells transfected with si-SREBPl (si-SREBPl-1 or si-SREBPl-2) or si-NC using western blot. (**d**) Intracellular triglyceride levels were detected in cells as decreased in **c**. *P<0.05.

The previous analysis showed that SREBPs (sterol regulatory element-binding proteins) were critical transcription factors that control lipogenesis and lipid uptake.^22^ SREBPs firstly located in endoplasmic reticulum membrane, which was considered as its inactive precursors. Once the sterol levels drop, SREBPs transferred from endoplasmic reticulum to Golgi apparatus, where mature forms were released by proteases (site-1 and site-2). Thereafter, mature SREBPs entered nucleus to bind SRE-containing gene promoters to induce transcription.^23^ Emerging evidence indicates that SREBP1 is a crucial linkage of oncogenic signaling transduction and cancer metabolism.^24^ To elucidate the molecular mechanisms of the TIP30 deficiency-mediated upregulation of lipogenic enzymes, cellular SREBP1 levels were examined. qRT–PCR and western blot analyses revealed that SREBP1 mRNA levels were significantly increased after TIP30 knockdown (Figures 1c and 2a). Additionally, immunostaining showed nuclear accumulation of SREBP1 with TIP30 deficiency (Figure 2b; Supplementary Figure 2B). Meanwhile, overexpression of TIP30 leaded to adverse effects on SREBP1 levels in HCC cells (Figures 1c and 2a). SREBP1 expression was reduced in TIP30 knockdown cells which TIP30 are re-expressed in (Supplementary Figure 2A). We then used small interfering RNAs (siRNAs) targeting SREBP1 to explore whether TIP30 deficiency upregulated lipogenic enzyme expression through SREBP1. SREBP1 depletion of TIP30-deficient HCC-LM3 and SMMC-7721 cells leaded to decreased expression of FASN and SCD (Figure 2c). Moreover, intracellular triglyceride levels and the intensity of BODIPY staining were remarkably reduced by siSREBP1 in TIP30-deficient HCC cells (Figure 2d; Supplementary Figure 2C). These results demonstrate that SREBP1 is involved in TIP30 deficiency related lipogenesis-promoting effects.

### Downregulating TIP30 enhances SREBP1 expression by activating the Akt/mTOR signaling pathway

**Figure 3:**
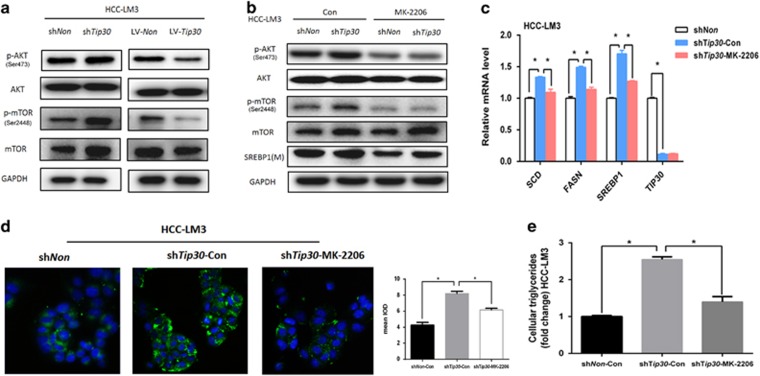
Downregulating TIP30 enhanced SREBP1 expression through activating Akt/mTOR signaling. (**a**) Western blotting showed p-AKT and p-mTOR levels of HCC-LM3 cells infected with sh*Non*, sh*Tip30*, LV-*Non* and LV-*Tip30,* respectively. (**b**) HCC-LM3-sh*Non* and HCC-LM3-sh*Tip30* were treated with AKT inhibitor MK-2206 (0.5 μm) for 24 h, indicated protein expressions were analyzed using western blot. (**c**) MK-2206 (0.5 μm) was added in HCC-LM3-sh*Tip30* cells. After 24 h of the treatment, mRNA levels of TIP30 and lipogenic enzymes were tested using qRT–PCR. BODIPY 493/503 staining (magnification, × 200) (**d**) and intracellular triglyceride levels (**e**) were performed in cells as decreased in **c**. For quantification of the mean integrated optical density (IOD) of BODIPY staining, Image J software was applied and values were analyzed with unpaired *t*-test. *P<0.05.

Loss of TIP30 can activate EGFR/AKT signaling of human lung adenocarcinoma and mammary cancer.^25, 26^ We have previously confirmed that TIP30 deficiency can activate AKT signaling in HCC and laryngeal carcinoma.^16, 20^ Previous studies have indicated activation of AKT/mTOR signaling had critical effects on lipid metabolism regulation.^27, 28^ Here, we find downregulation of TIP30 expression activates AKT and causes elevated mTOR phosphorylation in HCC-LM3 cells, whereas up-regulating TIP30 expression reduced AKT and mTOR phosphorylation levels (Figure 3a). Additionally, we found that blockade of AKT by its inhibitor, MK-2206, dramatically attenuated the TIP30 deficiency-induced up-regulation of p-mTOR and SREBP1 levels (Figure 3b). Moreover, MK-2206 could reverse the upregulated FASN and SCD expression in TIP30-deficient HCC-LM3 cells (Figure 3c). Intracellular triglyceride levels and the intensity of BODIPY staining were also reduced after MK-2206 treatment (Figures 3d and e). These data show that AKT/mTOR/SREBP1 signaling is required for TIP30 to regulate lipid metabolism in HCC cells.

### Decreased TIP30 promotes the proliferation of HCC cells via SREBP1-related lipid metabolism *in vitro* and *in vivo*

**Figure 4:**
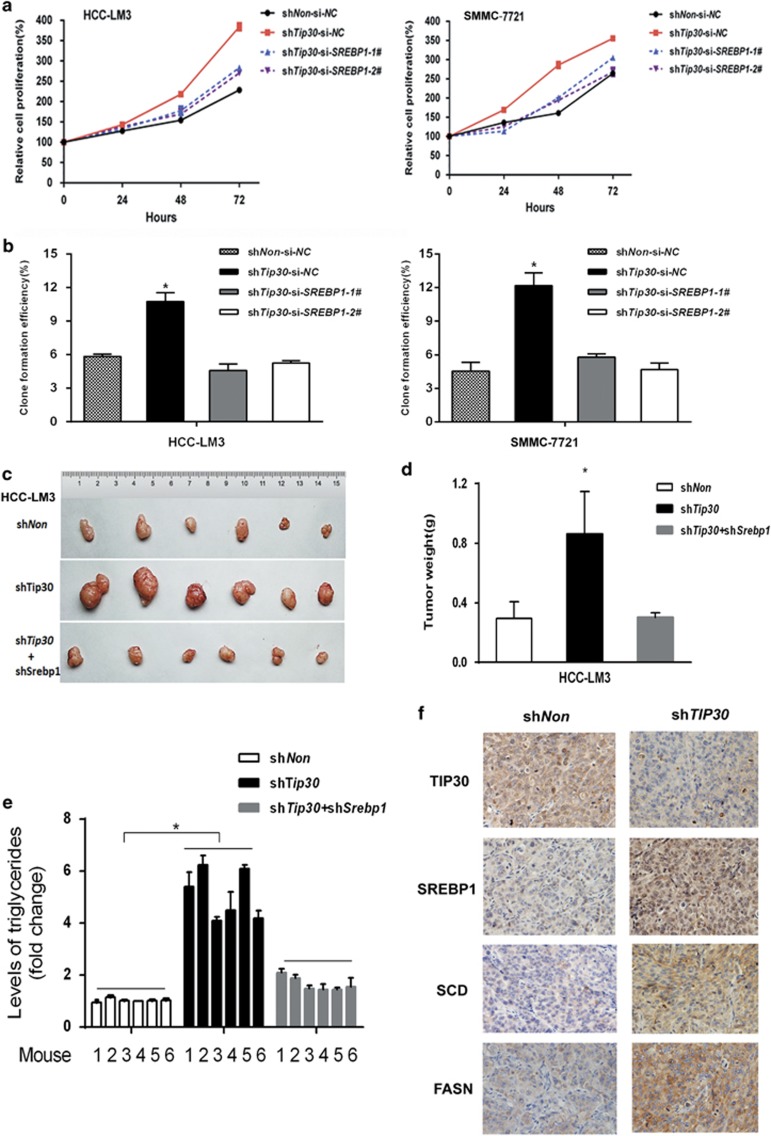
Decreased TIP30 promotes HCC cells growth *via* modulating SREBP1-related lipid metabolism. (**a**) Cell proliferation was tested in HCC-LM3-sh*Non* and HCC-LM3-sh*Tip30* transfected with si-SREBP1 (si-SREBPl-1 or si-SREBPl-2) or si-NC using MTS. Similar methods were repeated in SMMC-7721 cells. (**b**) Clone formation was generated in cells as decreased in **a**. (**c**) Pictures showed the tumors dissected from nude mice, which were transplanted with HCC-LM3 cells infected with sh*Non*, sh*Tip30*, or shTip30 and sh*Srebp1*. (**d**) Average weight of tumors was evaluated in each group. (**e**) Levels of triglycerides were individually measured in tumor tissues of each group using a tissue triglyceride assay kit. (**f**) Tumors derived from nude mice were immunostained for TIP30, SREBP1, SCD and FASN (magnification, × 200). *P<0.05.

Given important effects of lipid metabolism on tumor progression, we examined whether TIP30-regulated HCC cell growth via SREBP1-mediated lipid metabolism. As shown in Figure 4a, depletion of SREBP1 signaling with siRNA inhibited TIP30 deficiency-induced enhanced HCC-LM3 and SMMC-7721 cell growth. Using colony-forming assays, we found that decreasing the TIP30 deficiency-induced HCC cell colony formation was also dependent on SREBP1 (Figure 4b). To better understand the role of lipid metabolism reprogramming in TIP30-regulated HCC growth *in vivo*, xenograft studies were performed. We established stable HCC-LM3 cell lines with both TIP30 and SREBP1 deficiency and the corresponding cells were subcutaneously injected into 4 weeks old BALB/c nude mice. Results showed decreased SREBP1 significantly abolished the accelerated tumor growth of TIP30-deficient HCC-LM3 cells (Figures 4c and d). Interestingly, the levels of triglycerides were increased in the tumor tissues with reduced TIP30 expression, whereas silencing SREBP1 reversed this effect (Figure 4e), further supporting the conclusion that lipid metabolism deregulation contributed to HCC cell growth. Meanwhile, immunohistochemical staining showed tumors originated from TIP30-silenced HCC-LM3 cells exhibited increased SREBP1, SCD and FASN expression (Figure 4f), which was coincident with results obtained in HCC cell lines *in vitro*. Taken together, our results suggest that TIP30 can modulate SREBP1-related lipid metabolism, which contributes to tumor growth in HCC.

### Decreased TIP30 is associated with elevated SREBP1 levels in HCC samples, and combinational biomarkers provide powerful prognostic value for HCC patients

**Figure 5:**
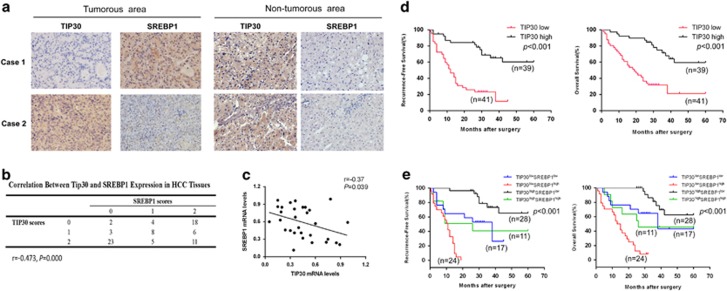
TIP30 expression negatively associates with SREBP1 level in tumor tissues of HCC patients and the combinational biomarkers provide powerful prognostic value for HCC. (**a**) TIP30 and SREBP1 were detected by immunostaining in 80 HCC samples. Representative pictures of immunostaining were shown for two patients. (magnification, × 200). (**b**) The correlation between TIP30 and SREBP1 expression was analyzed in HCC tissues according to the scores of immunohistochemistry staining. (**c**) Scatter plot showed correlations between TIP30 and SREBP1 mRNA level in tumors of 30 HCC patients investigated. Recurrence-free survival rates and overall survival rates were analyzed between TIP30-high expression group and TIP30-low expression group (**d**), as well as between four subgroups (TIP30^low^/SREBP1^low^; TIP30^low^/SREBP1^high^; TIP30^high^/SREBP1^low^; TIP30^high^/SREBP1^high^) (**e**).

To better understand the correlation between TIP30 and SREBP1 expression, immunohistochemical staining of 80 clinical HCC samples was performed. SREBP1 protein levels in HCC tissues negatively correlated with TIP30 expression (*r*=−0.473, *P*<0.001) (Figures 5a and b), suggesting that SREBP1 may be upregulated by TIP30 deficiency in HCC. We then measured TIP30 and SREBP1 mRNA levels in 30 HCC tissue samples investigated to provide further support for our research. Results showed the negative association(*r*=−0.37, *P*=0.039) between TIP30 and SREBP1 mRNA levels (Figure 5c). Using NCBI GEO databases to analyze the HCC sample array (GEO dataset accession GSE36376),^29^ we also found the negative correlation of TIP30 and SREBP1 in 240 HCC samples(*r*=−0.24, *P*<0.001), showing the same tendency as our results (Supplementary Figure 3). Meanwhile, patients with low TIP30 levels and high SREBP1 levels exhibited the poorest recurrence-free survival (RFS) as well as overall survival (OS), indicating that combinational detection of the two molecules may have a powerful prognostic value (Figures 5d and e).

## Discussion

Increasing evidences showed lipid metabolism was a key player in tumor growth, metastasis and resistance to therapies. As the main metabolic organ, liver is crucial for carrying out lipid metabolism, and aberrant activation of lipogenesis has been considered as an oncogenic event in human HCC.^30, 31^ In the present research, we evaluated whether TIP30 participates in abnormal lipid metabolism of HCC.

TIP30 was first discovered as a metastasis suppressor in 1997 and as a tumor suppressor in 2003.^6, 7^ The tumor suppressor function of TIP30 has been extensively demonstrated in various types of human tumors, including HCC. TIP30 exerts its tumor-suppressive role by influencing cell apoptosis, growth, metastasis and angiogenesis.^32^ Recently, TIP30 has also been confirmed to regulate the metabolic adaptation to glucose limitation of HeLa cells, which contributes to tumor metastasis and aggressiveness.^33^ For the first time, our study demonstrated that TIP30 is a negative regulator of lipid metabolism in HCC. We also demonstrated that decreased TIP30 may facilitate lipid metabolism through the AKT/mTOR/SREBP1 signaling pathway to promote tumor growth in HCC.

Normal tissues often utilize circulating lipids, while more than 90% of fatty acids are produced from *de novo* synthesis in tumors cells during their rapid growth and proliferation.^34^ Thus, several key lipogenic enzymes are activated to increase *de novo* lipogenesis of cancer cells. SREBP1-regulated downstream lipogenic enzymes (FASN and SCD), have been confirmed to be elevated in various tumors.^35, 36^ Consistently, our results demonstrated that TIP30 deficiency could promote the lipid synthesis of HCC cells via the up-regulation of FASN and SCD. We also found that the mRNA levels of two oxidative enzymes (CPT1A and ACOX1) were not affected by TIP30 in HCC cells. However, a recent report preliminarily suggested that TIP30-regulated fatty acid oxidations in normal hepatocytes by evaluating the CO_2_ production of cells labeled with [^14^C] palmitate.^21^ Considering the different cell lines and research methods applied in these data, the role of TIP30 in fatty acid oxidations of HCC needs further evaluation from transcriptional and post-transcriptional regulation.

As the main regulator of hepatic lipogenesis, SREBP1 is highly activated in cancers and activates the fatty acid pathway in human HCC cell lines. Genetic or pharmacological inhibition of SREBP1 resulted in cell growth arrest and decreased cell proliferation.^24^ Recent study reported that inhibition of *de novo* lipid biosynthesis by suppressing the SREBP pathway prevented HCC progression.^37^ Our present data also confirmed that decreased TIP30 could promote HCC cell growth via SREBP1-related lipid metabolism *in vitro* and *in vivo*, which was also responsible for elevated FASN and SCD expression induced by TIP30 deficiency.

Several experimental models have revealed critical effects of Akt on lipogenesis regulation. It has been recently found that liver tumors induced by AKT/c-Met displayed increased lipogenesis and genetic deletion of the main lipogenic enzyme, FASN, suppressed the *in vivo* hepatocarcinogenesis driven by AKT and c-Met oncogenes.^38, 39^ Another research reported that excessive activation of AKT in mice liver accelerated fatty acid synthesis as well as tumor development.^40^ In human lung adenocarcinoma, breast tumors and glioma, p-AKT and p-ERK1/2 were upregulated by TIP30 deficiency.^25, 26, 41^ We previously revealed that loss of TIP30 activated AKT/GSK-3β/β-catenin signaling, which was vital to growth, chemoresistance and self-renewal of laryngeal carcinoma.^16^ Additionally, downregulation of TIP30 could activate AKT to regulate the levels of epithelial–mesenchymal transition related transcription factors in HCC-LM3 cells.^20^ mTOR activation by Akt contributes to regulation of *de novo* lipogenesis. Through up-regulating SREBP1 transcription, processing and nucleic accumulation, mTOR signaling senses nutrients for growth and accelerates *de novo* lipogenesis.^42^ In particular, Calvisi has reported that AKT-mTORC1 signaling-induced lipogenesis accelerated HCC development from transcriptional and post-transcriptional aspects, including downregulation of FASN ubiquitination and interruption of SREBPs degradation.^40^ Consistently, in our study, SREBP1 was revealed to be upregulated by TIP30 deficiency-mediated Akt/mTOR activation. Meanwhile, CD147 has been reported to form a complex with integrinβ to activate PI3K/Akt pathway and then reprogram lipid metabolism through Akt/mTOR/SREBP1 signaling in HCC.^43, 44^ As a cancer-associated biomarker for detection and an effective target for treatment, CD147 also forms complexes with CD44 and EGFR to induces EGFR downstream signaling (ERK, pSTAT3) in breast cancer and pancreatic cancer.^45, 46^ Also, loss of TIP30 can improve EGFR activity in various tumors and Tip30 knockout in primary hepatocytes of mouse leads to trapping of EGF-EGFR complex, which contributes to prolonged EGFR signaling.^25, 26, 41, 47^ Considering both TIP30 and CD147 could regulate EGFR related signaling, it would be interesting to figure out whether downregulated TIP30 expression may synergistically act with increased CD147 expression in HCC in future research.

In addition, we confirmed that TIP30 expression was negatively associated with SREBP1 expression in clinical HCC samples. TIP30 is an important prognostic predictor for various cancers.^19, 48, 49^ Upregulated SREBP1 associated with a poor prognosis of HCC patients.^50^ Remarkably, when the combined effects of TIP30 and SREBP1 were evaluated, the sensitivity for survival analysis of HCC patients was improved.

In summary, we linked TIP30 to lipid metabolism through SREBP1 in HCC, which revealed alternative mechanisms underlying TIP30-induced growth regulation in hepatocellular carcinoma. Meanwhile, we also expanded the novel function of TIP30 in HCC metabolism regulation, which should be extensively studied in other types of tumors.

## Materials and methods

### Cell lines, antibodies and reagents

HCC-LM3 was supported by the Liver Cancer Institute of Zhong Shan Hospital (Shanghai, China). HepG2 was available from American Type Culture Collection. SMMC-7721 was acquired from Cell Bank of Shanghai Institutes for Biological Sciences (Shanghai, China). Cell lines have been tested for mycoplasma contamination and were cultured at 37 °C in a humidified condition with 5% CO_2_. The Dulbecco’s modified Eagle’s medium (DMEM) was used for cell culture, supplemented with 10% fetal bovine serum. Details of primary antibodies and their sources are as follows: TIP30 (produced by our lab as previously reported);^17^ p-mTOR (Ser2448), p-AKT (Ser473), mTOR, AKT, FASN and GAPDH (Cell Signaling Technology, Danvers, USA); SCD and SREBP1 (Abcam, Cambridge, USA). Additionally, we purchased horseradish peroxidase-conjugated secondary antibodies (anti-rabbit or anti-mouse) from Santa Cruz Biotechnology (SCBT, CA, USA). MK-2206 was from Selleck Chemicals (Houston, TX, USA). Oleate was acquired from Sigma (St Louis, MO, USA).

### Lentivirus and small interfering RNA

HCC cells were infected with lentivirus expressing *Tip30* cDNA or shRNA targeting *Tip30* as literature described.^17^ A siRNA targeting SREBP1 were designed by GenePharma (Shanghai, China). Briefly, the following sequences were used: for SREBP1-homo-523 (si-SREBP1-1), sense 5′-GCUCCUCUCUUGAAGCCUUTT-3′ and antisense 5′-AAGGCUUCAAGAGAGGAGCTT-3′ for SREBP1-homo-1403 (si-SREBP1-2), sense 5′-GCAACACAGCAACCAGAAATT-3′ and antisense 5′-UUUCUG GUUGCUGUGUUGCTT-3′.

### Protein extraction and western blotting

1 × SDS buffer was used to obtain total cell lysate. Equal amounts protein were loaded and then separated by SDS–PAGE. After the process of electrophoresis was finished, PDVF membranes were used for protein transferring from SDS–PAGE. After probing with primary and secondary antibodies, enhanced chemiluminescence’s reagents (Pierce Biotechnology, Milwaukee, WI, USA) were added to detect the antigen–antibody complexes.

### RNA extraction and real-time quantitative PCR

NucleoSpin RNA kit (Macherey-Nagel, Germany) was used for total RNA extraction. After the concentration of RNA was tested, reverse transcription PCR was done as the PrimeScript RT reagent Kit (Takara Bio, Tokyo, Japan) guided. SYBR Green-based real-time PCR was operated on a 7500 Fast Real-Time PCR System (Applied Biosystems, Carlsbad, CA, USA) and Gapdh was detected for endogenous control. Primer sequences are listed in Supplementary Table 1.

### BODIPY staining

HCC cells were fixed for 15 min using 4% paraformaldehyde. Thereafter, cells were washed then dyed by BODIPY 493/503 (10 μg/ml) (Thermo Fisher Scientific, Waltham, MA, USA) at 37 °C for 15 min.Then, the nucleus was counterstained with DAPI (Invitrogen, Carlsbad, CA, USA) for 10 min. We captured images using a fluorescence microscope (Olympus, Lake Success, NY, USA).

### TG measurements

The cellular TG content was tested using a Triglyceride Quantification Kit (Abcam, Cambridge, UK) with detailed experimental procedure provided in the kit.

### Immunofluorescence staining

HCC cells were incubated on 24-well plates for 24 h and then were fixed using paraformaldehyde with the final concentration 4%. Triton X-100 was prepared in PBS for the final concentration 0.2% and then was used for permeabilization.10% BSA/PBS was used as blocking buffer. For immunofluorescence staining, primary antibodies were added for incubation for 24 h on a shaker setting at 4 °C. After washing three times, a goat anti-rabbit Alexa Fluor 555 antibody (Thermo Fisher Scientific) was mixed and for 1 h incubation. Thereafter, cells were counterstained using DAPI (Invitrogen). All matched samples were photographed (control and test) using confocal laser scanning microscopy (FLUOVIEW FV-1000).

### Proliferation detection and colony-formation assays

The detached cells were incubated on 96-well plate (5000 cells/well). Proliferation was testified uasing MTS method (CellTiter 96 AQueous One Solution Cell Proliferation Assay, Promega Corporation, USA) at different time point as protocol guided. For colony-forming assays, single-cell suspension was prepared which was then seeded onto a 10-cm-diameter dish with 10 000 cells/dish. After 10 days, cultured cells were dyed by crystal violet for 15 min. Then, dye was washed out and we counted clones that were containing >50 cells. Clone formation efficiency was calculated as clones to total cells seeded on the dish.

### Microarray analysis

HCC-LM3 cell line was infected with sh*Tip30* or control lentivirus. After 7 days, TRIzol (Thermo Fisher Scientific) was used to extract cellular RNA, which was then purified by an RNeasy kit (Qiagen, Hilden, Germany). NimbleGen Gene Expression Microarray was applied in microarray analysis. Axon GenePix 4000B microarray scanner was used for scanning and raw data were extracted by NimbleScan software 2.5. Gene Ontology (GO) and Kyoto Encyclopedia of Genes and Genomes (KEGG) enrichment analysis of the differentially expressed genes (fold change ⩾2, *P*<0.05) were performed for predicting biologic effects of TIP30. Such analysis was achieved by the ClueGo plugin of Cytoscape (Software version 3.2.3, INSERM UMRS1138, Paris, France), which is a functional annotation way to evaluate over-representation of functional categories in interested genetic sets.^51^ Enrichment analysis was performed via functional annotation chart and annotation clustering options, which was limited to GO terms and KEGG pathways in ‘Biologic Process’ categories. Functional annotation was deemed significant with *P*-value<0.05, using Fisher’s exact test.

### Tumor xenograft mouse model

Animal studies are authorized by medical ethics committee of PLA General Hospital. Male Balb/c nude mice (4 weeks old) are randomly allocated into three groups (6 mice/group) and the number of mice is determined according to prior experience of *in vivo* studies in our laboratory. We subcutaneously inject 5 × 10^6^ indicated cells into each mouse. Investigators were not blinded for the animal studies. During the experiment, mice were monitored and euthanized for histopathology examination after cell inoculation for 28 days. Then, the tumor weight and their triglycerides levels were measured.

### Patients, immunohistochemistry and scoring

Samples of 80 patients who had radical resection of HCC were collected from 2003 to 2007 at Guangxi Cancer Hospital (Nanning, China). Radical surgery was defined as previously reported.^20^ Informed consent authorized by Ethics Committee of Guangxi Cancer Hospital was acquired from patients when specimen collection was performed. Supplementary Table 2 showed clinicopathological features of the above patients. All the patients were monitored for recurrence every 1–6 months after the curative resection depending on the post-operative time. Immunohistochemistry of clinical samples were performed as previously reported.^17, 20^ Two experienced pathologists independently evaluated the staining scores. According to the staining intensity and distribution, immunostaining scores were semiquantitatively estimated.^17^ Immunohistochemical scores of ⩽4 and scores of ⩾5 were classified as low and high expression, respectively.

### Statistical analysis

We repeated *in vitro* experiments in triplicate. SPSS software (version 16.0, Chicago, IL, USA) was used for statistical analysis. Pearson chi-square test and Student’s *t*-test were applied for analysis of dichotomous variables and continuous variables, respectively. Correlations of two variables were determined using Spearman rank test. Survival analyses of investigated patients were achieved using Kaplan–Meier analysis with log-rank test. When more than two data sets were analyzed, variance analysis was performed. The results are showed as the mean±s.e.m. The above statistical analyses were all two-sided with *P*<0.05 deemed statistically significant.
