# Supplementary figures and images for: TIP30 regulates lipid metabolism in hepatocellular carcinoma by regulating SREBP1 through the Akt/mTOR signaling pathway
Source: Oncogenesis. 2017 Jun 12;6(6):e347–. doi: 10.1038/oncsis.2017.49 (PMC5519197; doi:10.1038/oncsis.2017.49)

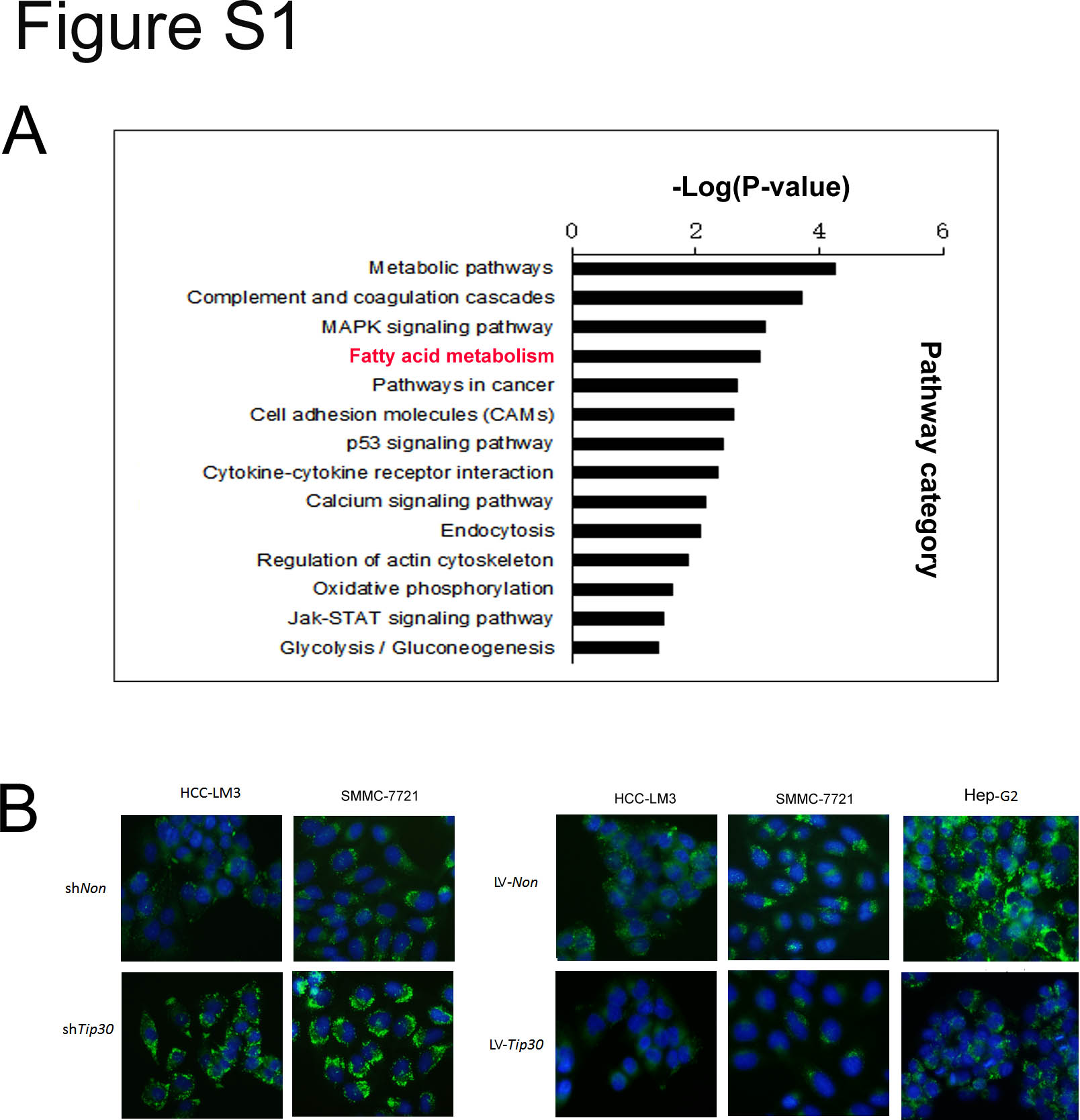

Supplement: Supplementary Figure S1 [file oncsis201749x1.tif]

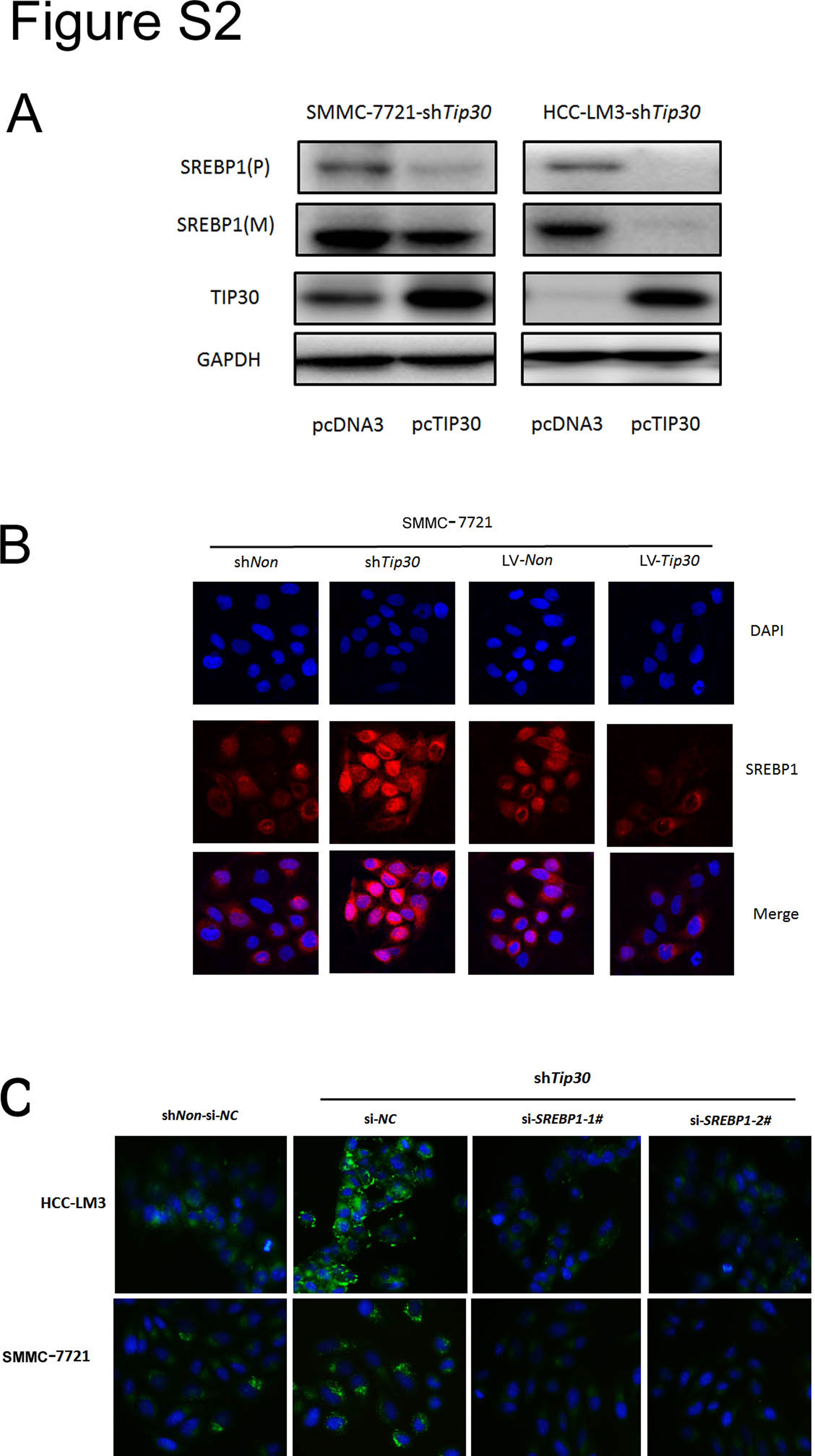

Supplement: Supplementary Figure S2 [file oncsis201749x2.tif]

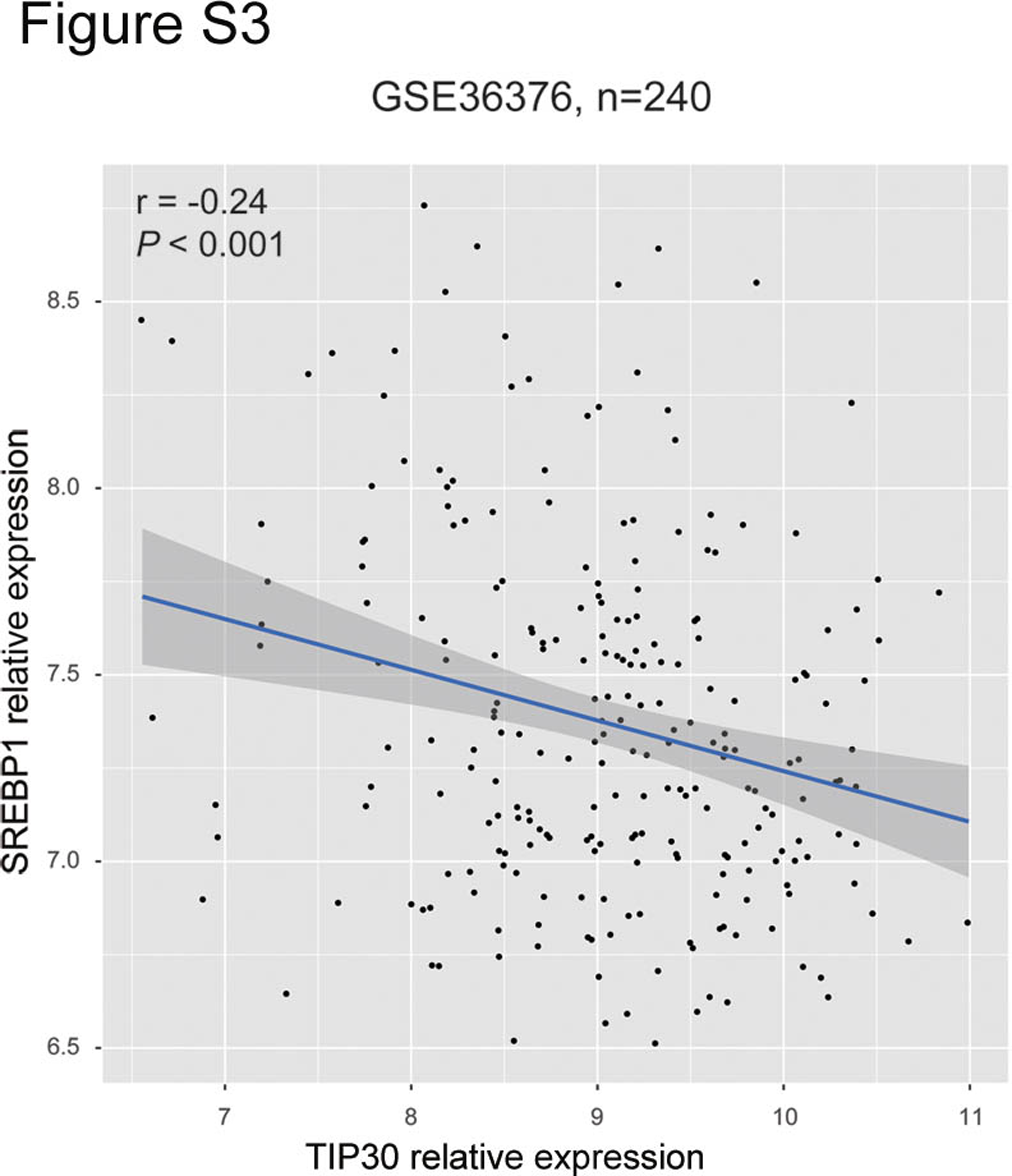

Supplement: Supplementary Figure S3 [file oncsis201749x3.tif]
