# Supplementary material for: TIP30 regulates lipid metabolism in hepatocellular carcinoma by regulating SREBP1 through the Akt/mTOR signaling pathway
Source: Oncogenesis. 2017 Jun 12;6(6):e347–. doi: 10.1038/oncsis.2017.49 (PMC5519197; doi:10.1038/oncsis.2017.49)
Supplement: Supplementary Figure Legends [file oncsis201749x4.doc]

**Supplementary Figure legends**

FigS1. TIP30 negatively regulates lipid metabolism in HCC cells.(A) Based on KEGG database, pathway analysis was performed to show canonical pathways regulated by TIP30 knockdown in HCC-LM3 cells, and pathways with significant changes are shown (*P* < 0.05). P value of each pathway was indicated by the bar and expressed as −1 times the log of P value.(B) Neutral lipids were stained by BODIPY 493/503 (green staining) in HCC-LM3 and SMMC-7721 cells with lipid accumulation inducer oleate (0.05mM) added in the culture medium. DAPI (blue staining) was used to stain nuclear. (magnification, ×200).

FigS2. TIP30 deficiency promotes lipogenesis through regulating SREBP1. (A) Western blot analysis on SREBP1 levels in HCC-LM3-sh*Tip30* and SMMC-7721-sh*Tip30* cells transfected with pcDNA3 or pcTIP30. (B) Results of immunofluorescence analysis were showed in indicated SMMC-7721 cells. Red staining represented SREBP1 protein. DAPI (blue staining) was used to stain nuclear. (magnification, ×200) (C) BODIPY 493/503 (green staining) was used to stain neutral lipids in HCC-LM3-sh*Tip30* and SMMC-7721-sh*Tip30* cells transfected with si-SREBPl (si-SREBPl-1 or si-SREBPl-2) or si-NC.DAPI (blue staining) was used to stain nuclear. (magnification, ×200).

FigS3. TIP30 and SREBP1 mRNA expression are negatively correlated in 240 HCC samples in NCBI GEO databases (GEO dataset accession GSE36376).
