# Supplementary material for: TIP30 regulates lipid metabolism in hepatocellular carcinoma by regulating SREBP1 through the Akt/mTOR signaling pathway
Source: Oncogenesis. 2017 Jun 12;6(6):e347–. doi: 10.1038/oncsis.2017.49 (PMC5519197; doi:10.1038/oncsis.2017.49)
Supplement: Supplementary Table [file oncsis201749x5.doc]

| **Gene symbol** | **Primer Sequence (5'-3')** |
| --- | --- |
| *TIP30* | F: TCACCTTCGACGAGGAAGCT |
|  | R: GCTCTGCAGACTTCAGACCA |
| *SREBP1* | F: GCCCCTGTAACGACCACTG |
|  | R: CAGCGAGTCTGCCTTGATG |
| *SCD* | F: TCTAGCTCCTATACCACCACCA |
|  | R: TCGTCTCCAACTTATCTCCTCC |
| *FASN* | F: AAGGACCTGTCTAGGTTTGATGC |
|  | R: TGGCTTCATAGGTGACTTCCA |
| *ACC* | F: CTTGAGGGCTAGGTCTTTCTGG |
|  | R: CTGGTTCAGCTCCAGAGGTT |
| *ACOX1* | F: ACTCGCAGCCAGCGTTATG |
|  | R: AGGGTCAGCGATGCCAAAC |
| *CPT1A* | F: TCCAGTTGGCTTATCGTGGTG |
|  | R: TCCAGAGTCCGATTGATTTTTGC |

**Supplementary Table 1.The primer sequences for real-time PCR**

**Supplementary Table 2. The clinicopathologic characteristics of 80 cases of HCC**

| **Variables** | **No.of patients(%)** |
| --- | --- |
| Gender |  |
| Male | 55 (69) |
| Female | 25 (31) |
| Age, *y* |  |
| ≤50 | 46 (58) |
| ＞50 | 34 (42) |
| HBsAg |  |
| Negative | 14 (18) |
| Positive | 66 (82) |
| AFP, *ng/ml* |  |
| ≤20 | 35 (44) |
| ＞20 | 45 (56) |
| Cirrhosis |  |
| No | 16 (20) |
| Yes | 64 (80) |
| Tumor size, *cm* |  |
| ≤5 | 47 (59) |
| ＞5 | 33 (41) |
| Tumor number |  |
| Single | 72 (90) |
| Multiple | 8 (10) |
| Vascular invasion |  |
| No | 70 (88) |
| Yes | 10 (12) |
| AJCC stage |  |
| Ⅰ-Ⅱ | 46 (58) |
| Ⅲ-Ⅳ | 34(42) |

Abbreviations: AFP, alpha fetoprotein; HBsAg, hepatitis B surface antigen; AJCC, American Joint Committee on Cancer
